# Long non-coding RNA: a new player in cancer

**DOI:** 10.1186/1756-8722-6-37

**Published:** 2013-05-31

**Authors:** Hua Zhang, Zhenhua Chen, Xinxin Wang, Zunnan Huang, Zhiwei He, Yueqin Chen

**Affiliations:** 1 China-America Cancer Research Institute, Key Laboratory for Medical Molecular Diagnostics of Guangdong Province, Guangdong Medical College, Dongguan 523808, China; 2 Key Laboratory of Gene Engineering of the Ministry of Education, State Key Laboratory for Biocontrol, Sun Yat-sen University, Guangzhou 510275, China

## Abstract

Emerging evidence showed that long non-coding RNAs (lncRNAs) play important roles in a wide range of biological processes and dysregulated lncRNAs are involved in many complex human diseases, including cancer. Although a few lncRNAs’ functions in cancer have been characterized, the detailed regulatory mechanisms of majority of lncRNAs in cancer initiation and progression remain largely unknown. In this review, we summarized recent progress on the mechanisms and functions of lncRNAs in cancer, especially focusing on the oncogenic and tumor suppressive roles of the newly identified lncRNAs, and the pathways these novel molecules might be involved in. Their potentials as biomarkers for diagnosis and prognosis in cancer are also discussed in this paper.

## Introduction

Genome-wide transcriptome studies have revealed that there exist a large number of non-coding RNAs (ncRNAs), including short and long non-coding RNAs [1,2]. Short ncRNAs have a length of under 200 nucleotides (nt) and include small interfering RNAs (siRNAs, 21-25 nt), piwi-associated RNAs (piRNAs, 24-33 nt) and well-documented microRNAs (miRNAs, 21-25 nt), while long ncRNAs (lncRNAs) are greater than 200 nt in length, frequently ranging up to 100 kb [3]. It has been known that lncRNAs, mRNA-like transcripts, are mainly transcribed by RNA polymerase II (RNA PII) and are polyadenylated, spliced, and mostly localized in the nucleus [3-5].

Recent studies estimated that the number of lncRNAs in humans is approximately 15,000 and found that most lncRNAs displayed tissue-specific expression patterns [6]. Based on their locations and characteristics, lncRNAs can be categorized into five subgroups: (1) sense, (2) antisense, (3) bidirectional, (4) intronic, and (5) intergenic [5]. Increasing studies have indicated that lncRNAs play important roles in a wide range of biological processes including stress response [7], development [8], embryonic stem cell pluripotency [9], localization [10], alternative splicing [11], chromatin remodeling [12] and mRNA decay [13]. Furthermore, lncRNAs can affect many cellular processes, such as cell cycle [14], survival [15], migration [16] and metabolism [17].

Because lncRNAs, like miRNAs, function as key regulators in gene regulation, it is not surprising that dysregulated lncRNAs are involved in many complicated human diseases including cancer. Mounting evidence showed that many lncRNAs, similar to miRNAs, have altered expression in various types of human cancer and dysregulated lncRNAs function as tumor suppressors or oncogenes [18,19]. Undoubtedly, lncRNAs have become new players in cancer after miRNAs although the detailed mechanisms of most lncRNAs remain largely unknown. In this review, we overviewed the roles of lncRNAs in tumorigenesis, focusing on the currently known mechanisms and functions of lncRNAs, and their potentials as biomarkers and targets for novel therapeutic approaches in the future.

### Regulatory mechanisms of lncRNAs

It has been known that ncRNAs function as a new class of regulators involved in a complicated molecular network, controlling gene expression and cellular activity. Among them, well-documented miRNAs were known to regulate gene expression at either the transcriptional or the post-transcriptional level usually by base-pairing to the 3′-untranslated region (3′ UTR) of target mRNAs with the miRNA 5′-proximal “seed” region (positions 2-8) in animals [20]. However, compared with miRNA, the regulatory mechanisms of lncRNA are more challenging to characterize, since these molecules are neither as conserved nor as abundantly expressed as miRNAs [21]. Recent studies have shown that lncRNAs regulate gene expression by diverse mechanisms.

#### Guiding role of lncRNAs in cis and in trans

Evidence showed that lncRNAs can bind and ‘guide’ chromatin modifying complexes to specific genomic sites to induce epigenetic changes and regulate gene expression *in cis* (on neighboring genes of the same chromosome) or *in trans* (on distantly located genes of the same or different chromosome) [22]. Several early discovered cis-regulatory lncRNAs were well-studied. For example, XIST represses genes on X chromosome by the recruitment of PRC2 (polycomb repressive complex 2) [23]; AIR and KCNQ1OT1 silence transcription of their target genes by recruiting G9a [24,25]; HOTTIP recruits the MLL histone H3 lysine 4 (H3K4) methyltransferase complex to maintain active chromatin [26]. LncRNAs can also regulate gene expression by guiding site-specific recruitment of chromatin modifying complexes *in trans*. It has been shown the lncRNA HOTAIR mediates the epigenetic repression by increasing PRC2 and LSD1 recruitment to the genomic positions of target genes [16]. In addition, PCAT-1, JPX and lincRNA-p21 function by trans-regulation of genes [27-29]. Interestingly, the lncRNA asOct4-ps5, an antisense to a pseudogene of Oct4, could regulate Oct4 by guiding the histone methyltransferase Ezh2 to the Oct4 promoter *in trans*[30].

#### Serving as scaffolds

Another regulation mechanism of lncRNAs is to serve as scaffolds. Studies demonstrated that lncRNA KCNQ1OT1 could bind both G9a and PRC2, and lncRNA HOTAIR could interact with PRC2 and LSD1, revealing these lncRNAs can serve as scaffolds for chromatin-modifying complexes besides their guide role [25,26]. Similarly, lncRNA ANRIL and NEAT1 were also found acting as scaffolds [31-33]. In addition, it was found that approximately 38% of large intergenic non-coding RNAs (lncRNAs) expressed in the cell types studied are reproducibly associated with chromatin-modifying complexes (PRC2, CoREST and SMCX) by RNA coimmunoprecipitation (RIP) with antibodies directed against several proteins involved in these complexes [34]. Thus, these studies implied that lincRNAs bind to chromatin modifying complexes first to serve as scaffolds, and then guide the complexes to specific genomic regions.

#### Transcriptional regulation

LncRNAs regulate gene expression at transcriptional level via multiple mechanisms. It was reported that by competing for binding sites, the lncRNA GAS5 (growth arrest specific 5) interacts directly with the DNA binding domain of glucocorticoid receptors (GR), preventing them from binding to their DNA response elements, thus functions in transcription inhibition as a molecular decoy [17]. In addition, recent studies revealed that novel classes of promoter-associated lncRNAs, the promoter upstream transcripts (PROMPTs) and enhancer-associated lncRNAs (eRNAs), are positively correlated with the level of messenger RNA synthesis and regulate the transcription activity of host protein-coding genes (PCGs) [35,36].

#### Alternative splicing and translational regulation

The well-known example of mRNA splicing mediated by lncRNAs is that lncRNA MALAT1 interacts with the serine/arginine-rich splicing regulatory (SR) proteins involved in alternative splicing, suggesting that the lncRNA MALAT1 may serve a function in the regulation of alternate splicing [11]. Another example of translational regulation for lncRNAs is that lncRNA BACE1-AS can interact with the β-site APP (amyloid precursor protein) cleaving enzyme 1 (BACE1) transcript and increase BACE1 mRNA stability, thus generate more gene product [37]. Notably, some pseudogenes have been found to regulate translation. For example, lncRNA pseudo-NOS, a pseudogene of nitric oxide synthase (NOS), can bind and repress translation of nNOS (neuronal nitric oxide synthase) gene through influencing the association of the ribosome with the nNOS–pseudo-NOS duplex [38]. Another representative mechanism is that some lncRNAs including pseudogenes, such as lncRNA PTENP1, a pseudogene of PTEN, serve as ‘sponges’ to prevent specific miRNAs from binding to their target mRNAs by competitively binding to miRNAs [39,40].

### Dysregulated lncRNAs in cancer

Transcriptional profiling has revealed highly aberrant lncRNA expression in human cancers [41]. Though the function of most lncRNAs remains unknown, accumulating evidence showed that differentially expressed lncRNAs are associated with cancer pathogenesis and function as new regulators in cancer development. These dysregulated lncRNAs in cancer are listed in Table 1. In this section, we mainly discussed their potentials as biomarkers, and their functions as oncogenes and tumor suppressors, as well as the molecular pathways they might be involved in.

**Table 1:** Examples of dysregulated lncRNAs in cancer

**lncRNA**	**Cancer type**	**Expression**	**Function**	**Reference**
MVIH	Hepatocellular	Upregulated	Biomarker	[42]
DD3 (PCA3)	Prostate	Upregulated	Biomarker	[43]
HOTAIR	Breast, hepatocellular, colorectal, gastrointestinal stromal	Upregulated	Biomarker	[16,44-47]
			Oncogenic	[16,48]
MALAT1	Lung, hepatocellular	Upregulated	Biomarker	[49-51]
	Lung, liver, renal cell, breast, cervical, uterine endometrial stromal, colorectal, bladder, osteosarcoma		Oncogenic	[52]
H19	Hepatocellular	Downregulated	Biomarker	[53]
	Hepatocellular, bladder	Upregulated	Oncogenic	[54]
PCAT-1	Prostate	Upregulated	Oncogenic	[27]
PCGEM1	Prostate	Upregulated	Oncogenic	[55]
TUC338	Hepatocellular	Upregulated	Oncogenic	[56]
BANCR	Melanoma	Upregulated	Oncogenic	[57]
YIYA	Hepatocellular, ovary, breast, esophageal	Upregulated	Oncogenic	[58]
CRNDE	Colorectal, hepatocellular, pancreatic, prostate, ovarian, leukemia, gliomas	Upregulated	Oncogenic	[59]
CCAT1	Gastric	Upregulated	Oncogenic	[60]
HULC	Hepatocellular	Upregulated	Oncogenic	[61]
CUDR	Bladder	Upregulated	Oncogenic	[62]
GAS5	Breast	Downregulated	Tumor suppressive	[63]
linc-p21	Mouse models of lung, sarcoma, lymphoma	Downregulated	Tumor suppressive	[29]
MEG3	Meningioma, glioma, hepatocellular, leukemia	Downregulated	Tumor suppressive	[64-67]
PTENP1	Prostate, colon	Downregulated	Tumor suppressive	[40]
PTCSC3	Papillary thyroid	Downregulated	Tumor suppressive	[68]

#### Serving as biomarkers for diagnosis and prognosis

Seeking novel molecular biomarkers of malignancy is always important and helpful for clinical diagnosis and management. Like proteins, mRNAs and miRNAs, lncRNAs show their potentials as novel independent biomarkers for early diagnosis and prognosis prediction in cancer.

It has been found that lncRNA HOTAIR is highly upregulated in breast cancer, hepatocellular carcinoma (HCC), colorectal cancer and gastrointestinal stromal tumors (GIST). Furthermore high HOTAIR gene expression is correlated with metastasis in these four types of cancers, poor survival rate in breast cancer, increasing risk of recurrence after hepatectomy in HCC and high-risk grade in GIST, indicating HOTAIR may be a useful biomarker of poor prognosis and tumor metastasis in these cancers [16,44-47]. In HCC, another lncRNA MVIH was found overexpressed and significantly associated with frequent microvascular invasion, higher tumor node metastasis stage, decreased recurrence-free survival (RFS) and overall survival, revealing that the upregulation of MVIH can serve as an independent risk factor to predict poor RFS [42]. More recently, it was reported that lncRNA H19 is underexpressed in intratumoral HCC tissues (T), as compared with peritumoral tissues (L), and low T/L ratio of H19 is associated with shorter disease-free survival and can be used to predict poor prognosis [53]. Studies also showed that lncRNA MALAT1 is upregulated and serves as an independent prognostic parameter for patient survival in early stage non-small-cell lung cancer [49,50]. LncRNA MALAT1 can also be used for predicting HCC recurrence after liver transplantation in HCC [51]. In prostate cancer, highly expressed lncRNA DD3 (PCA3) demonstrated better diagnostic efficiency than another promising biomarker telomerase reverse transcriptase (hTERT gene) for prostate cancer, and could be easily detected in prostate needle biopsies or bodily fluids such as blood, ejaculate, urine, or prostate massage fluid, thus lncRNA DD3 is a very sensitive and specific biomarker for the detection of prostate tumor cell [43].

Genome-wide association studies (GWAS) have revealed a large number of genetic variants related to diseases, including cancer. Recently, genetic variations in lncRNAs and their associations with cancer susceptibility have been reported, suggesting single nucleotide polymorphisms (SNPs) may also serve as biomarkers for diagnosis and prognosis. Studies showed that SNP rs2151280 in lncRNA ANRIL was significantly associated with higher number of plexiform neurofibromas (PNFs) in neurofibromatosis type 1 (NF1) patients, which suggests SNP rs2151280 in lncRNA ANRIL is a potential biomarker for PNF susceptibility [69]. Interestingly, it was found that the variant genotype of rs7763881 in lncRNA HULC is significantly associated with decreased risk to HCC in hepatitis B virus (HBV) persistent carriers, while variant rs10680577 within lncRNA RERT is a promising biomarker for early diagnosis of HCC [70,71]. Another recent study found that two genetic variations (rs6434568 and rs16834898) in lncRNA PCGEM1 might increase prostate cancer (PCa) risk [72].

#### Oncogenic and tumor suppressive lncRNAs

Like protein-coding genes and miRNAs, lncRNAs can function as oncogenes or tumor suppressors during cancer progression. A further investigation of the roles and mechanisms of lncRNAs in cancer will provide novel lncRNA-based strategies for the treatment of human cancers. Some well-studied lncRNAs, such as HOTAIR [16,48], MALAT1 [52], PCAT-1 [27], PCGEM1 [55], TUC338 [56], were reported as oncogenes, while GAS5 [63], linc-p21 [29], MEG3 [64] and PTENP1 [40] were reported as tumor suppressors. Here, we mainly focus on recently studied lncRNAs.

The newly identified lncRNA BANCR was found overexpressed in melanoma and required for full migratory capacity of melanoma cells by upregulating CXCL11, an important gene involved in cell migration, revealing the potential functional role of lncRNA BANCR [57]. Another novel lncRNA YIYA is upregulated in different cancers and promotes cell cycle progression at the G1/S transition, demonstrating it is a carcinogenesis-associated lncRNA [58]. A recent study found that lncRNA CRNDE is highly elevated in many solid tumors and in acute myeloid leukemias (AML), especially in M2 AML and M3 AML, and lncRNA CRNDE can promote cell growth and suppress apoptosis, which supports a role for CRNDE as a mediator of oncogenesis [59]. More recently, it was reported that lncRNA CCAT1, activated by c-Myc, is markedly increased in gastric carcinoma and promote cell proliferation and migration, implicating CCAT1 as a potential therapeutic target [60]. Studies also showed that induction of lncRNA HULC by Hepatitis B virus X protein (HBx) promotes proliferation of hepatoma cells through downregulating tumor suppressor gene p18 *in vitro* and *in vivo*[61]. In addition, it was found that lncRNA CUDR, a variant transcript of lncRNA UCA1, is upregulated in various human tumors and can significantly enhance proliferation, migration and invasion of the bladder cancer cell *in vitro* and *in vivo*, indicating lncRNA CUDR functions as an oncogene and may serve as a novel therapeutic target for bladder cancer [62]. Compared with these oncogenic lncRNAs, some lncRNAs showed a tumor suppressive role. The lncRNA MEG3 (maternally expressed gene 3), which is associated with meningioma pathogenesis and progression [64], was recently found markedly decreased in glioma tissues and ectopic expression of lncRNA MEG3 inhibited cell proliferation and promoted cell apoptosis via p53 activation in human glioma [65]. Very interestingly, a study reported that lncRNA PTCSC3 (Papillary Thyroid Carcinoma Susceptibility Candidate 3), located 3.2 kb downstream of SNP rs944289 at 14q.13.3, is strongly downregulated in papillary thyroid carcinoma (PTC). The overexpression of lncRNA PTCSC3 could inhibit PTC cell growth and affected the expression of genes involved in DNA replication, recombination and repair, cellular movement, tumor morphology, and cell death, implying that lncRNA PTCSC3 has the characteristics of a tumor suppressor [68]. Further study revealed that SNP rs944289 abolishes the binding site of both C/EBPα and C/EBPβ, which bind and activate the PTCSC3 promoter, thus, SNP rs944289 predisposes to PTC through downregulating tumor suppressive lncRNA PTCSC3 [68].

#### Pathways involving lncRNAs in cancer

Differentially expressed lncRNAs between cancer and normal tissues indicate these lncRNAs may exert a key role in cancer development. Understanding the pathways lncRNAs involved in helps to provide further insights into the pathogenesis of cancer. In HCC, PKA-mediated phosphorylation of CREB leads to formation of the CREB-p300-Brg I complex, loading of the CREB-p300-Brg I complex onto the lncRNA HULC promoter, and consequent activation of HULC expression [73]. In addition, lncRNA HULC can be activated by HBx via CREB protein and promotes proliferation of hepatoma cells through suppressing p18, an activator of p53 through its interaction with ataxia telangiectasia-mutated (ATM) in response to DNA damage [61,74]. These results suggest that lncRNA HULC also is involved in the ATM/P53 pathway in HCC. In bladder cancer, lncRNA UCA1 upregulates CREB activity through enhancing AKT activity, while blocking PI3-K pathway by PI3 Kinase inhibitor can downregulate lncRNA UCA1 expression and reduce cell cycle progression. These findings indicate lncRNA UCA1 promotes cell proliferation and regulates cell cycle progression through CREB via PI3-K/AKT dependent signaling pathway [75]. Another lncRNA CUDR may promote tumorigenesis through upregulating PDGFB in tumorigenesis pathway and downregulating FAS and ATM in cell apoptosis pathway in bladder cancer [62]. In addition, lncRNA profile and mRNA profile revealed significant changes in PPAR pathway in glioblastoma group compared with the normal group by target gene-related pathway analysis, such as ASLNC22381 and ASLNC2081 were found to play roles in glioma signaling pathways [76]. Moreover, the relative abundance of a collection of lncRNAs in pancreatic ductal adenocarcinoma (PDAC) was investigated, then intronic lncRNAs differentially expressed in PDAC metastases were enriched in genes associated to the MAPK pathway [77].

## Conclusions and perspectives

Recent studies revealed lncRNAs, like miRNAs, function as important regulators in gene regulatory networks and exert crucial roles in cancer progression. LncRNAs have become new and important players and demonstrated potential applications in clinical diagnosis and treatment. Although a few lncRNAs’ functions have been reported, there are still many challenges that remain to be solved. In the future, it would be necessary to further investigate the functional motifs and secondary or tertiary structure of lncRNAs, to fully elucidate the diverse gene regulatory mechanisms of lncRNAs, and to develop new and effective methods to predict target genes of lncRNAs using bioinformatics, etc. These endeavors will provide new insights into the complicated gene regulatory network involving lncRNAs, and ultimately provide novel strategies for cancer diagnosis and therapy.

## Competing interests

The authors declare that they have no competing interests.

## Authors’ contributions

All authors have contributed to write and revise the manuscripts. All authors have read and approved the final manuscript.
